# Implementation of a malaria prevention education intervention in Southern Ethiopia: a qualitative evaluation

**DOI:** 10.1186/s12889-022-14200-x

**Published:** 2022-09-23

**Authors:** Zerihun Zerdo, Sibyl Anthierens, Jean-Pierre Van geertruyden, Fekadu Massebo, Gelila Biresaw, Misgun Shewangizaw, Gesila Endashaw, Abayneh Tunje, Matewos Masne, Hilde Bastiaens

**Affiliations:** 1 Department of Medical Laboratory Science, College of Medicine and Health Sciences, Arba Minch University, Arba Minch, Ethiopia; 2 Global Health Institute, Antwerp University, Antwerp, Belgium; 3 Department of Family Medicine and Population Health, University of Antwerp, Antwerp, Belgium; 4 Department of Biology, College of Natural Sciences, Arba Minch University, Arba Minch, Ethiopia; 5 Department of Public Health, College of Medicine and Health Sciences, Arba Minch University, Arba Minch, Ethiopia; 6 School of Nursing, College of Medicine and Health Sciences, Arba Minch University, Arba Minch, Ethiopia

**Keywords:** Malaria prevention education, Qualitative, Context specific factors, School-aged children, Ethiopia

## Abstract

**Background:**

Though school-aged children (SAC) are at high risk of malaria, they are the ones that benefit the least from malaria prevention measures. A cluster randomized controlled trial was conducted to evaluate the effect of malaria prevention education (MPE) on insecticide-treated bed net (ITN) utilization and prompt diagnosis, reported incidence and treatment (PDAT) of malaria. Qualitative evaluation of the implementation of such interventions is vital to explain its effectiveness and will serve as guidance for future interventions. Therefore, this study aimed to evaluate the implementation of the MPE in southern Ethiopia.

**Methods:**

The trial was registered in Pan African Clinical Trials Registry (PACTR202001837195738) on 21/01/2020. A descriptive qualitative study using semi-structured interview with participants of the MPE was conducted in January 2020 and January 2021. The collected data were transcribed verbatim and analyzed thematically. The analysis of the data was supported by NVivo.

**Results:**

The four themes identified after evaluation of MPE training were the setup of the training, challenges for the success of the training, anticipated challenges for practice as per the protocol and experienced immediate influences of the training. Participants appreciated the training: content covered, way of delivery and the mix of the participants. The context specific facilitators to bed net use were the collateral benefits of ITN and perceived at high risk of malaria while its barriers were quality and quantity of the bed nets, bed net associated discomforts, malaria health literacy and housing condition. Severeness of malaria symptoms and malaria health literacy were reported as both barriers and facilitators of the PDAT of malaria. The identified facilitators of PDAT of malaria were health professionals’ attitude and exposure to MPE while its barriers were poverty, use of traditional medicine, health facility problems and Coronavirus Disease 2019 (COVID-19) pandemic.

**Conclusion:**

Low attendance of parents in the training was the major challenge for the success of MPE. National malaria program should ensure the access to malaria prevention measures; and future studies using increased frequency of the intervention embedded with monitoring adherence to the intervention protocol shall be conducted to improve the gains from existing malaria interventions.

**Supplementary Information:**

The online version contains supplementary material available at 10.1186/s12889-022-14200-x.

## Introduction

Malaria is one of the leading causes of morbidity and mortality among infectious diseases in the world. It is caused by a protozoan parasite of the genus *Plasmodium* and transmitted through the bite of female *Anopheles* mosquito. Except *Plasmodium(P). knowlesi,* the other four Plasmodium species causing malaria are found in Ethiopia; but *P. falciparum* and *P.vivax* are almost all causes of malaria in Ethiopia [1, 2]. Globally, there were an estimated 241 million malaria cases and 627, 000 deaths in 2020 of which 95% of the cases and 96% of the deaths occurred in the World Health Organization (WHO) African region [3]. The WHO and the world malaria community envisioned to see malaria free world [4]. The mainstays of malaria prevention measures are vector control strategies and prompt diagnosis and treatment (PDAT) of malaria cases by artemisinin-based combination therapy (ACT) [5]. In October 2021, RTS, S is declared by the WHO as the 1^st^ effective malaria vaccine to be used together with the child vaccination program in moderate or high malaria endemic settings [3].

Because of the intensified malaria prevention measures undertaken by the national malaria control programs and partner organizations, a remarkable achievement was made in the last two decades. Between 2010 and 2015, malaria cases and deaths were reduced by 21% and 31% respectively in the WHO African region [2, 5]. Long-lasting insecticide-treated bed net use by children age under-five reduced the mortality rate by 18.8% as revealed from Ghana Demographic and Health survey [6]. However, low exposure of children under-five years of age to malaria parasites lead to delayed development of antimalarial immunity. Consequently, school-aged children (SAC) have become another group at high-risk to contract malaria. A study from Malawi revealed that SAC were as equally susceptible as under-five children and more susceptible than pregnant women to malaria. In Addition to the increased susceptibility, SAC serve as the major reservoir for Plasmodium parasites; the gametocyte carrier state is multiple folds higher among SAC than the general population [7].

SAC have been severely affected by malaria and associated morbidities. About 200 million SAC are living in malaria transmission settings in SSA [8]. About 50% of these children were infected by Plasmodium species and malaria was accountable for 50% of SAC mortality [9]. A recent survey undertaken in Malawi indicated that the prevalence of malaria was significantly higher among SAC (34.8%) than children younger than 5 years (28.5%) and adults age above 15 years (27.3%) [10]. A nationwide survey undertaken in Côte d'Ivoire also revealed that the prevalence of malaria among this population subgroup was 73.9% [11].

Though SAC play the major role in the transmission of malaria and their increased risk of morbidity and mortality, they were not given due attention in the malaria control programs. They most often visit informal drug shops for the management of their clinical malaria and the least benefited from bed net use [7, 12, 13]. Olapeju and his colleagues assessment of age and gender trends in the disparity of bed net use in SSA demonstrated that the bed net use was sinusoidal. It was observed to be the lowest among SAC as compared to children age less than five years and women of the childbearing age [14]. In Malawi where the prevalence of malaria peaks among SAC, SAC less likely sought malaria treatment from formal sources (government or private clinics) as compared to their under-five counterparts [15].

Efficient and effective utilization of malaria prevention measures is dependent on the level of awareness [6, 16]. Misperception and misconception towards malaria was the major barrier for bed net utilization among pregnant women and under-five children in northwest Ethiopia [17]. Knowledge about the transmission of malaria was the major risk factor of bed net utilization in Northern Gonder, Ethiopia [18]. Similarly, our previous study in kutcha district in southern Ethiopia also shown that there is misperception of cause of malaria by parents of SAC which significantly influenced their malaria prevention practices [19]. For this reason, well-tailored malaria prevention education is recommended for correcting such misconceptions, misperceptions and improve awareness about malaria to increase the benefits from malaria prevention measures [20–23].

How and where such malaria prevention education should be implemented was not well documented. To address this gap, MPE package was developed and evaluated using a cluster randomized controlled trail in randomly assigned schools in southern Ethiopia [24]. The rationale for this choice is being that school-based anthelmintic drug distributions for soil-transmitted helminthiasis and schistosomiasis are proven to be effective as schools pool SAC in the same compound [25]. This paper focusses on the qualitative evaluation that was embedded alongside the trial in order to explore the experiences of the participants (both implementers and the target group), anticipated challenges for practice as per the protocol and the context specific factors affecting actual implementation of insecticide-treated bed net (ITN) utilization and PDAT of malaria, outputs of the trail. This information can be used to explain the outcomes of the trial and can offer guidance to improve the implementation of similar interventions in the future.

## Methods

### Study area and intervention

**Fig. 1:**
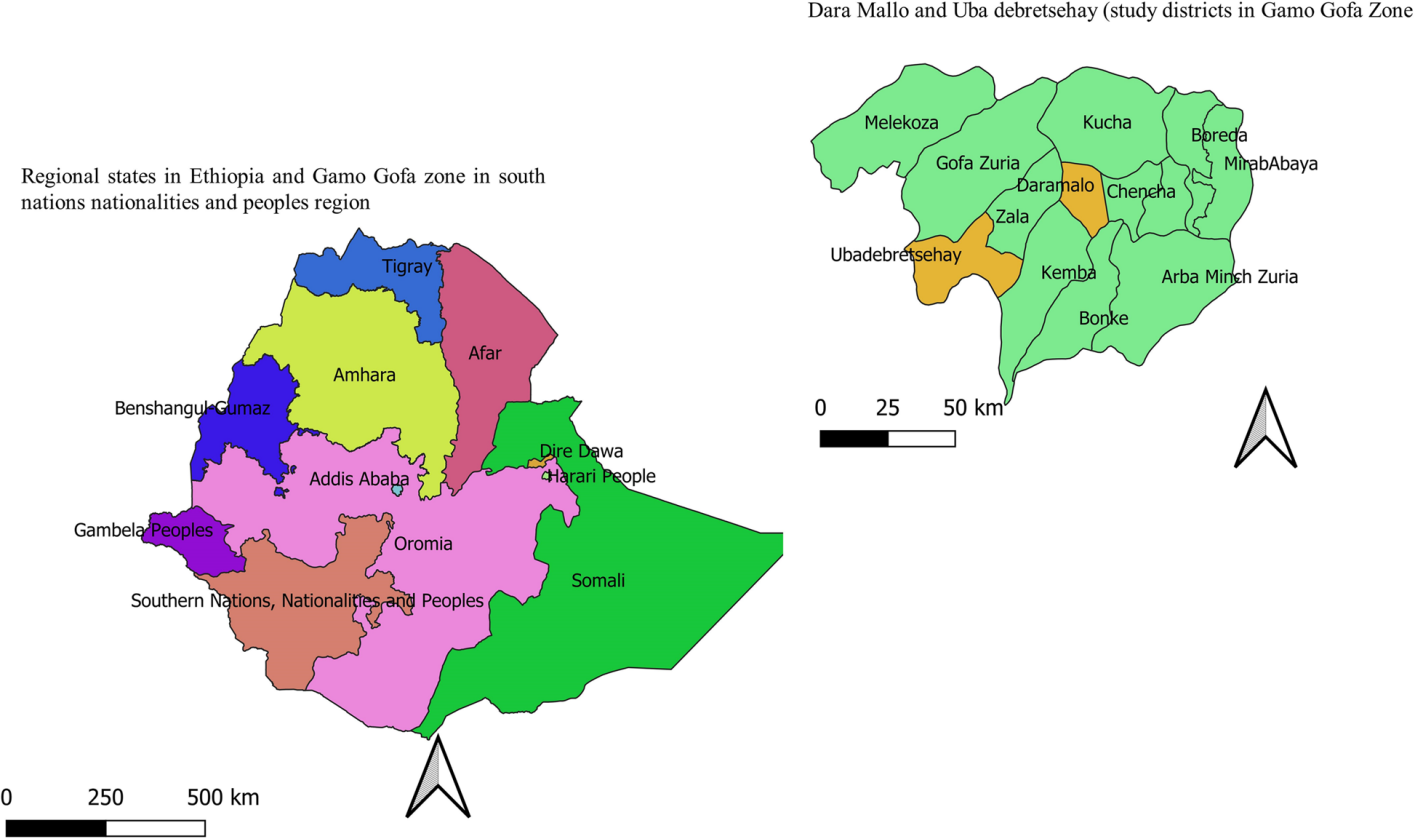
Location map indicating where the present study was conducted in southern Ethiopia, 2020–2021

This study was conducted in Dara Mallo and Uba Debretsehay Districts in Southern Ethiopia. According to the 2007 national census, a total of 150,145 people were living in the two districts, and of these 76,550 (51%) were males [26]. The updated population in the study area was described in the previous article [27]. An update made by the respective districts in 2020 indicates that there was a total of 94,396 people in Uba Debretsehay district and 110,207 people in the Dara Mallo district. The location map of the study area was indicated in Fig. 1.

The intervention, MPE, was developed after exploring the parents’ perception of cause of malaria and their malaria prevention experience among SAC in kutcha district in southern Ethiopia [19] and existing literature. The intervention is designed to correct misperception of cause of malaria and incorrect malaria prevention practices. The intervention providers (trained science teachers and school headmasters from each school) first explored the perception of cause of malaria and their malaria prevention experiences. Then, the intervention providers discussed the correct causes of malaria and malaria prevention measures from the MPE document. This document included topics on the cause of malaria, consequences of malaria, prevention of malaria, symptoms of malaria and treatment of malaria. In addition, both the children as well as their parents were demonstrated on how to properly fix the bed nets. The intervention is given to the SAC (5–14 years) and their parents in separate groups by the trained science teachers from each school.

The trial was registered in Pan African Clinical Trials Registry with the registration number of PACTR202001837195738 on 21/01/2020. This intervention was implemented, in the last week of December 2019, by trained science teachers and school headmasters in the intervention schools. As indicated above, the implementers started the training by exploring the perceived causes of malaria and their lived experiences, and then corrected the misperceptions and incorrect experiences. We hypothesized that improved knowledge on the right cause of malaria and consequences of malaria coupled with increased awareness about malaria prevention strategies could lead to effective and consistent utilization of malaria prevention strategies. Knowledge of the symptoms of malaria and awareness about the consequences of malaria would make parents to seek medical care for their child from the health facility promptly. Prompt diagnosis and treatment would interrupt the onward transmission of malaria parasites to other susceptible individuals and malaria associated morbidities and its complications.

### Study design, sampling and data collection

A descriptive qualitative evaluation was used to address the research questions. First, we explored how the participants experienced the intervention, immediate influences of the intervention and anticipated challenges for practice as per the protocol of the intervention. In the 2^nd^ time point of data collection, the context specific barriers and facilitators of the ITN utilization and PDAT of malaria were explored.

### Sampling for evaluation of the training and anticipated challenges for practice as per the intervention protocol

A total of 25 participants (9 intervention implementers and 16 recipients) were included in the study to address how the participants experienced the intervention and anticipated challenges to practice as per the intervention and immediate influences of the intervention. They were sampled based on the maximum variation sampling technique. Those involved in the study were diverse with respect to district, residence place, the quality of the intervention (as described by the setup of the training delivered), gender, and the role in the intervention process (either intervention implementer or parent of the selected SAC).

### Sampling for barriers and facilitators of ITN utilization and PDAT of malaria

At the end of the trial, we explored the barriers and facilitators for ITN utilization and PDAT (the outcomes we focused on in the intervention) of malaria (implementation during the trail) and looked at how the intervention assisted participants. To explore the barriers and facilitators of ITN use by SAC, all participants involved in the study were eligible; but for PDAT of malaria, only children who had shown symptoms of malaria after the intervention were included.

Twelve parents of the SAC and the 9 key informants were involved to address the barriers and facilitators of the ITN utilization and PDAT of malaria. The key informants recruited were the intervention providers, health extension workers (government paid females trained on the health extension program), malaria focal persons in the district health offices and health centers in the study area. These were also sampled by using the maximum variation sampling technique.

### Method of data collection

The data collection, using semi-structured interviews, was done at two time-points. The first one, addressing the evaluation of the training, was conducted about two weeks after the intervention. The topic guides were developed with the main focus of the big themes mentioned above: how the intervention participants experienced the intervention, its immediate experienced influences and the anticipated challenges to practice as per the protocol.

The 2^nd^ time point was one year after the intervention when context specific factors affecting ITN utilization and PDAT of malaria were explored. The topic guides were developed with the main focus of the sub-themes mentioned: barriers, facilitators and how the intervention influenced the ITN utilization and PDAT of malaria.

The parents were interviewed in their residence homes while the school directors were interviewed in the school compound or their residence area. Interview with the health extension workers, malaria focal persons in the health centers and district health officers were held in their respective working offices. Participation in the intervention was monitored by independent monitors, who were not among the research team to avoid bias. Information from this independent monitoring was used to select the participants of the intervention and its quality.

There were three individuals who carried out the interview process. They were health professionals with MSc in maternal and child health, MPH in health education and promotion and MSc in tropical and infectious diseases. All interviewers have experience of qualitative research and conducting interviews. The interviews were audio recorded and notes were taken during the data collection process. The interviews were undertaken in local languages (Gamogna or Gofegna) and Amharic (commonly spoken language all over Ethiopia) depending on the preference of the participant in the study. The interviewers involved in the data collection process were fluent in the local languages and Amharic (commonly spoken language in Ethiopia).

### Data analysis

After each interview, there was a debriefing session with the interview team in order to adapt the topic guide when necessary to improve data collection. The digital audio material was transcribed by one of the three interviewers in verbatim. The transcribed data were translated to english and read once again and the data analyst listened repeatedly to the audio material in order to deeply immerse into the data.

The data were analyzed thematically by ZZ (one of the researchers involved in the interview process and trained in qualitative research methods)-always keeping the main focus of the big themes and subthemes mentioned above. These were used as a guiding frame. First the initial transcripts were coded line by line to unravel the data. After 4 interviews, these open codes were segregated/ordered based on their similarities into subthemes. These subthemes are further grouped and refined to form big themes and discussed among the research team. The analysis of the data was supported by the new QSR NVivo version 1.5.1 (940) [28]. At the different stages of the analysis, the codes, subthemes and themes were discussed with the other two senior experts in qualitative research (HB; SA).

## Results

### Qualitative evaluation of the training (MPE)

Twenty-five participants (9 trainers and 16 trainees) were interviewed for qualitative evaluation of MPE: 18 men, 7 women, 17 in rural and 8 from urban area’s and their characteristics were described in Additional file 1.

**Fig. 2:**
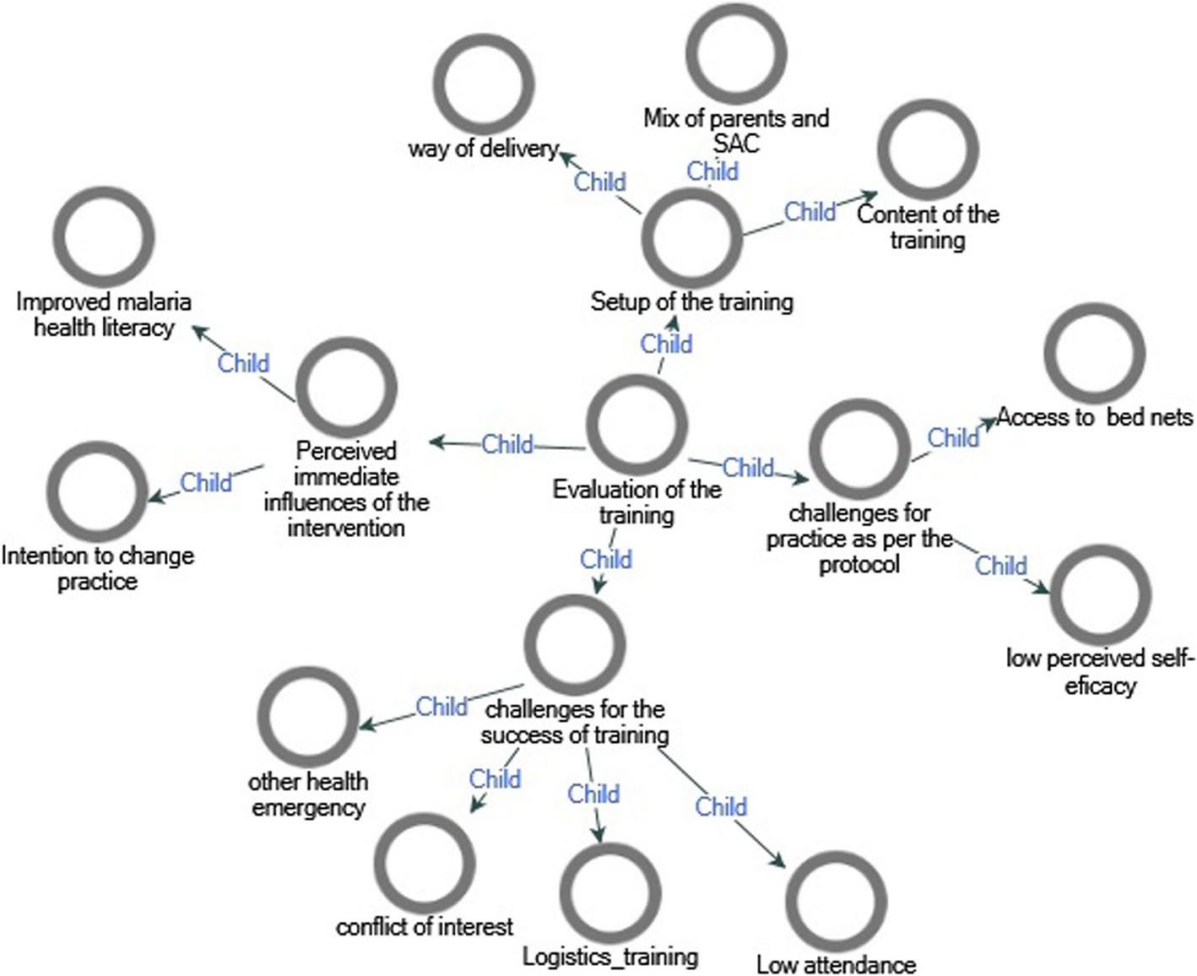
Themes and subthemes of evaluation of MPE training in southern Ethiopia, 2020

Four big themes were identified after the analysis of the data on how the participants experienced the training. These are the setup of the training, the challenges for the success of the training, anticipated challenges for practice as per the protocol of the trial and immediate perceived influences of the training (Fig. 2).

### Setup of the training

The participants appreciated the content and its coverage: exploring the perceptions of the cause of malaria and corrections made, the way of transmission and how malaria can be prevented.

“The training was good; it was about how to prevent malaria, cares to be taken, ways of transmission and cause of malaria”-[IM7].

“Language, active participation, correcting misconception and misuse of bed net are good”-[ IM2].

As for the way of delivery participants liked that the training was given in a quiet, disturbance free environment; the training being delivered by well experienced teachers in comprehendible manner and with active participation of the trainee. They also appreciated the mix of participants (both SAC and their parents) involved in the training.

“All students were in classroom except those who are called for the training, so there was no disturbance”- [ IM7]. “The trainer’s education was very nice, really very nice because he was not too hurry; he delivered education slowly in suitable way. In fact, there was no disturbing situation”-[ PAR1]. “Everyone asks and got answer for all questions. We have got the training in this way”-[IM7].

#### Challenges for the success of the training

The main challenge mentioned was the low attendance that was attributable to several reasons. Some of the parents come to the school late, others hurry to go back for their regular activities, and still some others missed the training at all because of overlapping meetings in the kebele, government employees for their office jobs in the town, weak communication and other regular duties. The parents were sensitized to come to the school for the training by the trained teachers and the selected children. However, the participants argued that communicating the participants through kebele leaders and the village leaders could increase the participant numbers.

“During the training, some people come late, others were absent due to mud in the road and raining as it was raining season”-[IM5].

“As this is town, most people are government employee, merchant and daily laborer. Due to this people responded lately as they are unable to participate. Some others made phone calls to inform that they were unable to participate”-[IM2].

Other challenges were logistics related factors. Participants reported that note taking materials for literate individuals and coffee and tea service provision would reduce the numbers hurrying to go back to their home and wait patiently for those coming late for the training.

“If training room, notebook, coffee and tea are considered, it would be good”-[PAR6].

“what do you say about the need of coffee, tea and others? [Interviewer]. If these were available it would be good. It would help people to sit long time-[IM7].

In one of the schools conflict of interest between the trainer and the headmaster of the school made the training to be started late.

A final challenges for the success of the training was other health emergency**.** In few of schools, the training was delayed due to public health emergency; children did not resume the regular school activities soon after the control of the emergency.

“There is other public health emergency in our community- even schools were closed for couple of days. After schools are opened, students were not coming to school”-[IM1].

#### Anticipated challenges for practice as per the protocol of the trial intervention

Two main challenges for implementing the intervention as indicated in the trial protocol were access to the bed nets and perceived low self-efficacy to conceptualize and use the malaria prevention measures. The participants reported that the bed nets that they have were not adequate to prevent their children from malaria.

“The main issue is not on the content, but we need bed net. Cause, manifestation and prevention of malaria were the contents of the training. These were good content those we need”- [PAR6].

The study participants perceive that they were not able to conceptualize the training given at once and need repeated training and follow-up.

“As we are farmers, we may forget it. For this reason, we need the training to be delivered repeatedly”-[PAR8].

#### Perceived immediate influence of the training

The immediate positive influences reflected by the participants after the training were improved malaria health literacy and intention to change practice. The participants reported that their knowledge on transmission of malaria and the consequences of untreated malaria was improved. Improved malaria health literacy changed participants intention to practice. It was mentioned that the sleeping behavior, sleeping anywhere before the dinner than under the bed net, was seen as bad behavior to be improved to protect children from the bite of mosquitoes.

“Yeah! The strong side was that it was all about malaria. If one of the family members is sick of malaria and not treated early, he/she will transmit it to rest of the family members. Malaria is lethal if not treated, so early treatment and malaria prevention were the messages delivered in the training. I consider this as strong side of the training”-[PAR4].

“I said successful because before the intervention people did not want to have bed net, but after the training was given, they want even to buy for themselves. I think people accepted the message”-[IM6].

“Before dinner children sleep outside the bed net; the only time they sleep under bed net was after dinner which is not good practice. So, the training clarified such ambiguities”-[ PAR6].

### ITN use and PDAT of malaria

Consistent Insecticide-treated bed net (ITN) use and prompt diagnosis and treatment (PDAT) of malaria are the output of the trial that the participants should adhere to reduce malaria among SAC. A total of 12 parents of SAC (8 with history of malaria during the follow-up and four without history), five malaria focal persons from the five health centers in the two districts, two directors from the intervention schools and two health extension workers were involved in the interview. The mean age of the participants was 36.6 years with the standard deviation of 6.5 years and 14 of the participants were male in gender (Additional file 2).

**Fig. 3:**
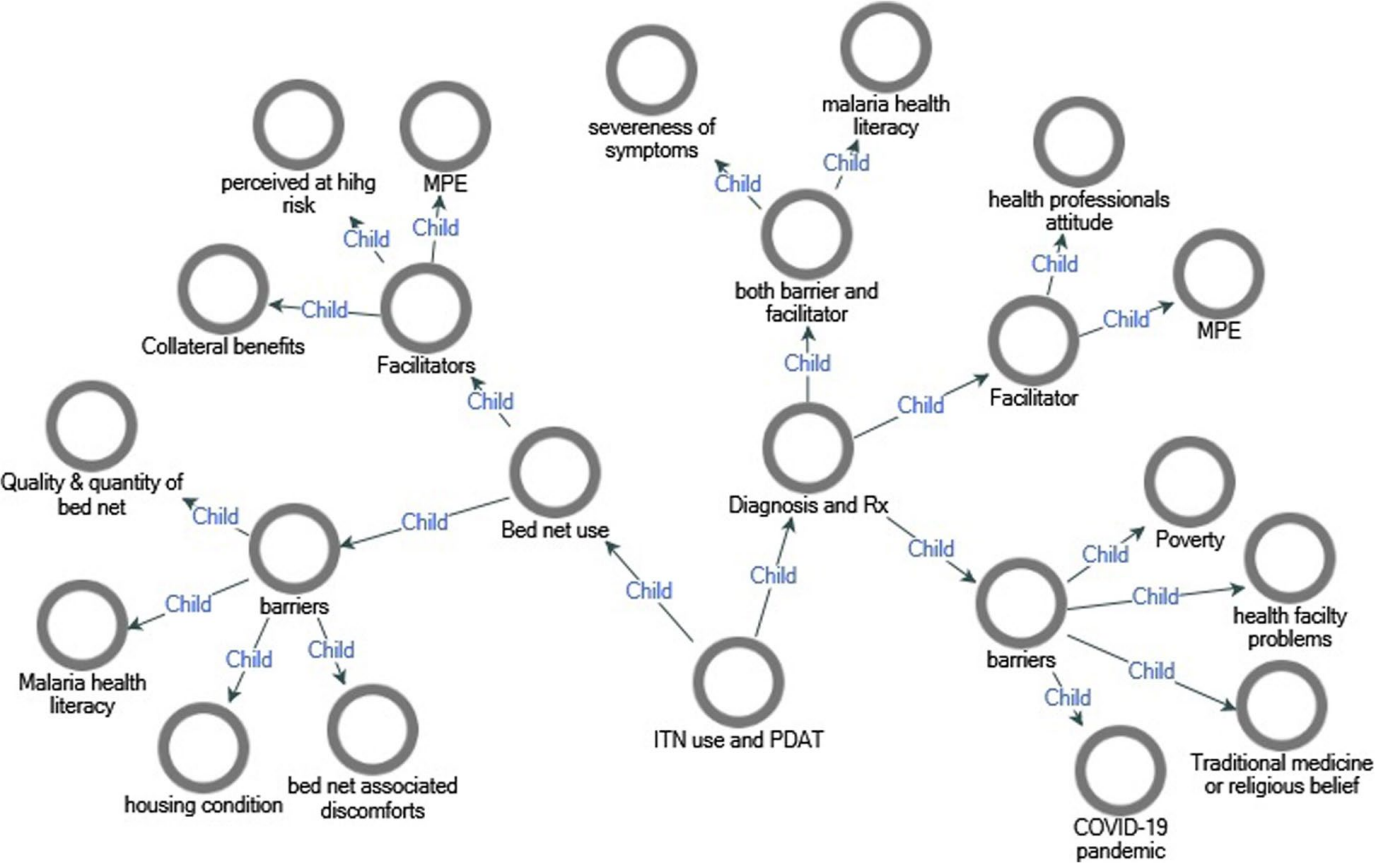
Themes and subthemes of barriers and facilitators of ITN use and PDAT of malaria in southern Ethiopia, 2020

There are different themes identified from the analysis of interview with the parents on context specific factors affecting the outputs of the intervention (Fig. 3).

### ITN use

Four barriers (quality and quantity of bed net, malaria health literacy, housing condition and bed net associated discomfort) and three facilitators ( perceived at high risk of malaria, collateral benefits and MPE) to ITN were identified.

### Barriers to ITN utilization

Participants reported that the warmth inside the bed net was not comfortable to sleep under it. In addition, they view that the bed net attracts the bed bugs, mainly when it gets dirty or not clean and in hot temperature. It was perceived that the hot temperature triggers the bed bugs to be placed on the bed net and become nuisance for sleeping.

“…If it (bed net) becomes dirty, the bed bugs enter into the bed net and become problematic” – [PAR4].

“When the environment becomes hot, the bed bugs came out from where they are hidden. In cold conditions though the bed bugs were there on the bed, they do not came out.” -[PAR1].

“Now the time is sunny. As a result, the bed net is in its rolled place and he(participated SAC) is not using it” [PAR10].

The 2^nd^ important barrier for bed net utilization by SAC was housing condition where they are living. This theme was identified from interview with the key informants. In some large household sizes, it is noted that the area of the house was not adequate to have the required number of sleeping places to properly fix the bed nets. In such conditions, children sleep together in a single temporary make-up bed over which a bed net is fixed; but it did not remain in the fixed place for the whole night.

“In some of the households with larger household size, the area of the house is narrow to have adequate number of sleeping places. As a result of this, people are not using bed nets properly or timely”-[KEY7].

The other housing condition believed to affect the proper utilization of the bed net was the structure of the houses, particularly in rural area. The traditional houses in the rural area were observed as not suitable to fix the bed nets unlike the homes made of corrugated iron sheet with its own sleeping bed rooms in urban areas.

“In the rural areas where the house type is traditional, it is difficult to properly use the bed nets in the rural areas. If the houses are made of corrugated iron sheet with its own bed room, it is convenient to use the bed nets” – [KEY6].

The respondents perceive that there was variation among the participants regarding malaria health literacy. It was felt that those educated people living in the urban area could better adhere to malaria related information delivered than those farmers residing in the rural areas.

“Those who had formal education use the bed nets as instructed by the health professionals”- [KEY6].

The other point was the belief that it is supernatural power that can protect their children from malaria.

“They say it is God that keeps them safe from attack of malaria and they pass the night outside the bed nets”- [KEY7].

Another notion related to the low malaria health literacy was taking the IRS as a replacement of bed nets in houses where it is sprayed and children being given low priority in houses where the bed nets are inadequate for the household members. Some people also appear to be unaware of the importance of using bed nets on a regular basis. These people were aware that mosquitos might spread malaria, yet they were ignorant to use bed nets even after detecting mosquitos in their home.

“Sometimes, even in the presence of mosquitoes, we do not use bed net due to ignorance”- [PAR3].

“Sometimes, if there is spray inside the house, we do not use bed net. During these times, we do not see mosquitoes. The spray destroy the mosquitoes, as result we do not use bed net”- [PAR3].

The other barrier to bed net use is bed net quantity and quality. In case of shortage of bed net, SAC use bed nets only when they share sleeping places together with the prioritized ones or the prioritized ones were not around home.

“The selected SAC did not pass the night under the bed net when the number of children in the household are high. At that time, the selected child passes the night together with her mother. However, when her big brothers went to farm area to keep their agriculture products from wild animals, she passes the night under bed net”-[PAR4].

The size of the bed net and the perceived low protective efficacy of the insecticide impregnated to the bed nets are qualities described as barriers for be net utilization.

“The previous bed nets are strong; it kills anything that lands on it. However, the current bed nets were not strong in killing. The previous one attracts like electric does and kills all insects. A mosquito that makes sound in the house cannot escape the killing of the previous bed net. Thus, we need such bed nets” – [PAR7].

### Facilitators of ITN utilization

The participants mentioned three main facilitators to bed net use by the SAC. The 1^st^ one is the observed collateral benefit of the bed net. The bed nets, specifically when the chemical impregnated was strong, destroys all insects those land on the bed net. This, as expressed by the participant, protects the individual sleeping under the bed net from the annoyance of such insects.

“If the bed net is new, it also kills the house flies. The fly that lands on the bed net gets died because the bed net is with the treatment. It also benefits us in this way”-[PAR10].

The 2^nd^ facilitator of bed net use by the SAC was perceived increased risk to malaria. Participants felt that children with repeated attack of malaria should use the bed nets consistently. In addition, they perceive that they were at high risk to acquire malaria due to the seasonal abundance of mosquito and being resident in the lowland area.

“We are using the bed net to protect the entry of mosquito. The mosquito number becomes very high during torche ( local term for summer season)”-[PAR9].

“They use bed nets when they are repeatedly affected by malaria”- [KI7].

Finally, the training they received also supported bed net use. As to the interview with the school director and the parents of SAC, the SAC were seen as actively participating in protecting themselves from malaria by using the bed nets.

“Since he is taught that the children should sleep under the bed net, we hang the bed net properly as instructed during the training. Those who go to the lowland area also take the bed net with them and protect from malaria by using bed net”-[PAR10].

“After following the last malaria prevention education in this school, children were using bed nets in a better way. I am exposed to this while moving from house-to-house for the different reasons”- [KI7].

After the intervention, the participants stated that their malaria health literacy has improved. Their impression of the protective efficiency of bed nets improved, as seen by their restricted usage of bed nets to protect from mosquito bite. Prior to the intervention, participants indicated that malaria was caused by a variety of factors other than mosquito bites.

“It gave us knowledge to care about our children to prevent from malaria”- [PAR5].

“Previously, we take bed net and use it for maize-to expose to sun light. We did this because of poor awareness we have regarding bed net utilization. Now, we are using the bed net only for the protection from bite of mosquitoes”-[PAR6].

In addition, they gained practical skills during the training in using the bed nets and applied it at home, like properly fixing the bed nets. Furthermore, their acceptability of other malaria prevention interventions like spray of the IRS chemical is improved; they were more actively engaged in malaria prevention; and they participate in the dissemination of their knowledge to the neighbors.

“Previously the way how we use the bed net is not like how we are using after the training. In this year, we gave due emphasis to the place where the children pass the night” –[PAR5].

### Prompt diagnosis and treatment (PDAT) of malaria

Severeness of malaria symptoms and malaria health literacy influenced the health seeking behavior of parents. How parents judge the severeness of the symptoms influenced whether they would go to a health facility; and this is perceived to be linked to how knowledgeable they were. If the parents view that their child was showing symptoms of sever malaria such as cerebral malaria, called as “*bicha woba*” in local language, they soon took the sick child for the treatment to avoid death or absenteeism from the school.

“We take soon to the health facility in fear of bicha woba (cerebral malaria). This malaria is placed in the brain” - [PAR11].

“Unless the child is bed ridden and severely affected, we do not take him to the health facility”-[PAR5].

“I did not spend much time after noticing the symptoms of malaria. I prefer to be diseased than my child being diseased”- [PAR11].

“We are rural people that might be the reason”. “What is the difference between people in the urban and rural area?” [interviewer]. “In the urban area, there are people who are more educated, they take the child to the health facility immediately”. “How about the rural?” [interviewer]. Now we are also improving as compared to the previous behavior. Thus, it is lack of knowledge”-[PAR6].

### Facilitators of PDAT of malaria

The perceived attitude of the health professionals was another facilitator. They believe that the health care provider diagnosing the child would be angry if the child was presented to the health facility after long duration after onset of malaria symptoms.

“If we delay for longer time, the health professionals in the health center become angry up on us. For this reason, we took her soon to the health center”- [PAR8].

The participants also expressed, that the MPE influenced their behavior in taking a sick child sooner to the health facility.

“Previously, before the training, we may delay up to one month. Now, we wait only up to two days to take the child to the health facility”- [PAR6].

“How MPE training influenced in treatment seeking? [ Interviewer]. “Yes, it benefited us. It benefited us to use antimalaria drugs from the health facility” [PAR8].

### Barriers to PDAT of malaria

Poverty was seen as a barrier for PDAT of malaria. The interviewees stated that poor socioeconomic position and lack of cash in rural dwellers' wallets were the identified reasons for the delay in malaria diagnosis and treatment. The other point of view was that once a SAC is diagnosed with malaria in a health institution, they self-medicate against malaria based on previous experience**.** Furthermore, it was thought that the malaria their children were suffering from should not be familiar with contemporary treatment because such drugs would be required during all the time their child was suffering from malaria. These viewpoints also seem to stem from a perception of being unable to afford the cost of healthcare.

“We are farmers. When the child says, aba (common name for fathers in Ethiopia), I am suffering from headache. Since we do not have money in our pocket, we should look for harvesting our agricultural product to sell. For this reason, we wait for some time hoping that it will be resolved spontaneously”- [PAR5].

“If malaria becomes familiar with modern treatment, it will not respond to this treatment in the future or may also always need that treatment. Tomorrow there will be a time in which we may be in shortage of money, at that time we may face problem if we make the disease familiar with the modern medicine” -[KI7].

The other factors that contributed to the delay in PDAT of malaria were health facility problems: lack of staff, private pharmacies selling antimalaria drugs without prescription and unavailability of drugs in the health centers. Because of these issues at the health facility and lack of a rule requiring parents to obtain a prescription before purchasing antimalarial drugs for their sick children, parents purchase antimalarial drugs without getting a proper diagnosis.

“First, we went to the health post. There is no person to diagnose and treat. Then, we went to the pharmacy and bought antimalarial drug and gave it to her” -[PAR5].

“Some others also purchase drugs from outside and give it to their children. Without any investigation, they take drugs from pharmacy. If the child is previously diagnosed as malaria case, they suspect the next cause of disease as malaria too. Thus, they buy antimalarial drugs from private pharmacy. If I take the child to health center and diagnose by giving blood, it would not be different from the previous disease”- [KI6].

Use of traditional or homemade remedies and religious beliefs were the other reported barriers for PDAT of malaria. People who were strong in their religious beliefs prefer praying for the child by the church leaders than taking their children to the health facility. Some others perceive that the symptom of malaria will go away by the traditional medicine or homemade remedies.

“There are certain traditional medicines which we used. it is …, garlic, fenugreek seed and others to treat malaria” -[PAR5].

**“**Some do not take the child to the health center. Rather, they wait for the symptom to go away by itself. We will not take the child to the health center. We should take the child to church to pray by the religious leaders”- [KI6].

The last barrier was COVID-19 pandemic. People were afraid of COVID-19 in the early stages of the pandemic, which caused a delay in the diagnosis and treatment of malaria. They conceived that COVID-19 was a disease of health professionals since it was health workers who were first infected by the virus. Fear of contracting the virus from a health institution or quarantine, and a sense that they are unable to adhere to COVID-19 preventative measures such as wearing a face mask, were among the other concerns mentioned.

“The health facility does not allow us to enter the health facility without a face mask. We do not have access to face mask because this is a rural area. Thus, I came back to home”- [PAR12].

“Due to COVID-19, for some weeks, even malaria patients do not come to the health center. This is because of fear of acquiring COVID-19 since about 15 of our staff were diagnosed to be infected by the virus”- [KI4].

“We measure the temperature of those visiting health center. This created fear that they will be quarantined. You cannot easily enter into this health center; rather, you are expected to wash hands, use hand sanitizer and get your temperature taken to screen for COVID-19. They do not know as sample should be collected to diagnose COVID-19”- [KI12].

## Discussion

This study aimed to qualitatively evaluate the MPE given to children and their parents in order to make the SAC to consistently use bed nets and get PDAT from the health facilities. Parents involved in the MPE training appreciated the setup of the training. Low attendance of parents, other health emergency, conflict of interest and some logistics were the reported challenges for the success of the training. Perceived low self-efficacy and inadequate access to the bed nets were the anticipated challenges to put the learnings into practice. Inadequate access and poor quality of the bed nets, bed net associated discomfort and housing condition were the barriers to bed net utilization by SAC while its facilitators were collateral benefit, perceived high risk to malaria and participating in the training offered in the trial. The severeness of malaria symptoms and malaria health literacy were reported as both the barrier and facilitator of PDAT. The other identified barriers to PDAT were poverty, problems of the health facility, COVID-19 pandemic and traditional medicines or the religious beliefs while attitude towards the health professionals and participating in the MPE were reported as facilitators.

One of the challenges for the success of MPE was the number of SAC parents presented to the training in the trial. This is problematic for the reach of the information expected to be delivered to all the parents of the children and would have a negative influence on the outputs of the training. Future studies shall discuss the best route of communication and date when such interventions should be held with the kebele leaders, leaders of the village and school headmasters. In addition, it is important to increase the frequency of MPE in order to better improve the malaria health literacy; and eventually to improve the participants self-efficacy to adhere to the malaria prevention measures and avoid the misuse of bed nets. It was seen that the low malaria health literacy and resulting wrong perceptions [29], being unaware of the purpose of the bed net [30], lack of knowledge of connection between bed net utilization and malaria [31], misuse of the bed nets or viewing the other malaria prevention measures such as IRS as replacement of bed nets were the threats for the bed net utilization in Ethiopia and other countries [29, 32–34].

Malaria health literacy was also barrier to PDAT of malaria. This finding was in agreement with a finding from a survey undertaken in Dare district in northwest Ethiopia where people with good awareness about malaria and its prevention measures soon sought treatment from the health facility [35]. In southwest Nigeria, mothers of febrile pediatric children perceived that malaria is simple disease; and they consequently used homemade herbal medicine for the treatment of malaria [36]. This was also observed in northwest Nigeria and the upper west region of Ghana where homemade remedies and herbal medicines were used to treat malaria [37, 38].

The perceived high risk of malaria and collateral benefits of the bed nets, mainly avoiding nuisance of flies facilitated the consistent bed net utilization by the SAC in this study. These added benefits of consistent utilization of bed net can be used to increase the bed net utilization in future malaria messages. These were also described in previous studies. In the Lake Tana area in Northern Ethiopia, killing arthropods other than mosquitoes was expressed as facilitator for bed net utilization [29]. Bed net utilization among under-five children in Zanzibar was influenced by its capacity to protect the nuisance of the mosquitoes in seasons when mosquito number was high [34]. Repeated attack by malaria, seen as confirmation for increased risk of malaria, was viewed as a factor facilitating bed net use in Dar es Salaam [33] and in Ghana [31].

The bed net associated discomforts such as warmth inside the bed net and infestation of bed bugs was the reported barriers to bed net use in this study. It was also corroborated by a study in the Lake Tana area (Northern Ethiopia) [29] and Zanzibar [34]; the same was also true in Ghana [31]. Therefore, it is important to consider chemicals effective in removing the bed bugs from the households. In addition to the warmth inside the bed nets, the study participants complained about adequacy of bed nets for their household members, its size and efficacy of the insecticidal chemical impregnated with it. The size of the bed net was seen as a reason for un-tuck of the bed net during the nighttime in Dar es Salaam [33]. The bad quality of bed net reported mainly from participants in the rural area was the bed net being not convenient to fix in the sleeping places of their common traditional home. This was also exhibited in the Lake Tana area [29]. Preference on the bed net quality should be studied and communicated to bed net producing companies to enhance its consistent utilization.

The severeness of malaria symptoms were reported as a factor affecting PDAT of malaria. Presentation of severe malaria symptoms was observed to be facilitator to treatment seeking behavior in a tertiary health facility in Nigeria [39]. These symptoms happen due to delay with the diagnosis of malaria; and, the outcomes of treating such cases were not satisfactory [40]. Before visiting the health facilities, parents might use other options such as homemade herbal medicines or purchase drugs from informal shops. However, the outcome of treating sever malaria forms, specifically cerebral malaria was significantly associated with child mortality [37].

The major barriers for seeking medical care from the health facility were associated with poverty and the health facility related problem. The economic problem as a barrier to the use of health service utilization indicates the importance of integrating economic development strategies as one of the malaria preventive measures. Bing in the lowest social class or women in the polygamous relationship in Nigeria [36, 39, 41], financial problems in Lao people’s Democratic republic [42] and in River region in Zambia [43] were factors impeding health seeking behavior for the treatment of malaria. Financial problem was also mentioned as a reason for using herbal medicine and leftover drugs [38]. Unlike these, a secondary data analysis of the national malaria indicator survey in Mozambique indicated that those above the poor categories as compared to the poorest did not significantly affected PDAT of malaria[44].

The health facility related barriers to the treatment of malaria were due to human power, diagnostic and treatment shortages and attitude towards the health facility. This needs urgent action to avoid such problems by the national malaria program in order to realize the prevention and elimination targets of malaria. In Nigeria, perceived bad attitude towards the health care providers and absence of health personnel in the health facility were considered as an impediments of care seeking from the health facilities [36, 41]; which was the case in Lao people’s Democratic republic [42]. The other health facility related problem for PDAT of malaria in this study area was a shortage of antimalarial drugs in the health facility while its presence in the private drug sellers where it should not exist in principle. Like this study, treatment seeking from the health facility was hindered by informal drug sellers in Vietnam [45]. Such drug sellers were the source for inadequate self-treatment of malaria in Nigeria [41]. The presence of antimalarial drugs in the private pharmacies and taking medication without proper diagnosis may lead to undertreatment of malaria and subsequent drug selection and resistance by malaria parasites. Thus, the national malaria control program should investigate the situation, take corrective measures and ensure rational use in order to save the life of the drugs.

Finally, there is a strength and a limitation associated with this study and those reading this paper shall take these into account while interpreting the findings. The strength was the study area being mostly rural and hard-to-reach while its limitation was not providing the intervention repeatedly as planned due to the COVID-19 pandemic.

## Conclusions

Low attendee was the major challenge for the success of MPE intervention. To improve the attendance of the trainee and avoid overlapping activities in the community, future studies shall consider the kebele, village and school leaders on the date and the best possible route of recruiting participants for the intervention. The barriers for consistent bed net utilization were discomforts associated with the use of bed net, the housing condition, mainly in the rural areas being not convenient to fix the bed nets, low malaria literacy, and quality and quantity of the bed nets. However, collateral benefits of bed net and perceived at high risk of malaria were the facilitators of bed net use.

Severe malaria symptoms and malaria health literacy were reported as both barrier and facilitators of PDAT of malaria. Poverty, health facility problems, use of traditional medicine or strong religious belief and COVID-19 pandemic were the identified barriers to PDAT of malaria while attitude towards the health care providers and exposure to the training in the trial were its facilitators. The malaria control program should ensure adequate access to malaria prevention measures and stringent policy of restricting the accessibility of antimalarial drugs at informal drug vendors should be there to maintain proper and effective use of antimalarial drugs. This shall be augmented by free laboratory diagnosis of malaria like its treatment or health insurance coverage should reach 100% to avoid the irrational use of antimalarial drugs or delay in the diagnosis of malaria. Implementation research of repeated malaria prevention education with an embedded process evaluation will be important to realize maximum benefit from existing malaria prevention measures.

## Supplementary Information

**Additional file 1.** Demographic characteristics of participants involved in exploring how participants experienced the intervention and anticipated challenges for practice as per the trial protocol.

**Additional file 2.** Socio-demographic characteristics of participants involved in the interview of barriers and facilitators of ITN use and PDAT of malaria, 2020.

## Data Availability

All data generated or analyzed during this study are included in this manuscript.

## Abbreviations

WHO: World Health organization
SAC: School-aged children
MPE: Malaria prevention education
PDAT: Prompt diagnosis and treatment
ITN: Insecticide-treated bed net
COVID-19: Coronavirus Disease 2019
ACT: Artemisinin-based combination therapy
IRB: Institutional Research Ethics Review Board
PAR: Participant
IM: Implémenter
KI: Key informant
