# Supplementary material for: Implementation of a malaria prevention education intervention in Southern Ethiopia: a qualitative evaluation
Source: BMC Public Health. 2022 Sep 23;22:1811. doi: 10.1186/s12889-022-14200-x (PMC9508754; doi:10.1186/s12889-022-14200-x)
Supplement: Supplementary file 1 — Additional file 1. Demographic characteristics of participants involved in exploring how participants experienced the intervention and anticipated challenges for practice as per the trial protocol. [file 12889_2022_14200_MOESM1_ESM.docx]

Additional file 1: Demographic characteristics of participants involved in exploring how participants experienced the intervention and anticipated challenges for practice as per the trial protocol

| **ID** | **District** | **Participant type** | **Age** | **Sex** | **Place of residence** |
| --- | --- | --- | --- | --- | --- |
| IM1 | Uba | Implementeer | 47 | Male | Urban |
| IM2 | Uba | Implementeer | 34 | Male | Urban |
| IM3 | Uba | Implementeer | 28 | Male | Ruar |
| IM4 | Uba | Implementeer | 30 | Male | Ruar |
| IM5 | Dara | Implementeer | 37 | Male | Ruar |
| IM6 | Dara | Implementeer | 30 | Female | Semi |
| IM7 | Dara | Implementeer | 28 | Female | Semi |
| IM8 | Dara | Implementeer | 32 | Male | Ruar |
| IM9 | Dara | Implementeer | 42 | Male | Urban |
| PAR1 | Uba | Recipant | 41 | Male | Ruar |
| PAR2 | Uba | Recipant | 39 | Male | Urban |
| PAR3 | Uba | Recipant | 37 | Male | Ruar |
| PAR4 | Uba | Recipant | 26 | Female | Urban |
| PAR5 | Uba | Recipant | 29 | Male | Ruar |
| PAR6 | Uba | Recipant | 40 | Male | Ruar |
| PAR7 | Uba | Recipant | 47 | Male | Ruar |
| PAR8 | Dara | Recipant | 35 | Male | Ruar |
| PAR9 | Dara | Recipant | 34 | Male | Ruar |
| PAR10 | Dara | Recipant | 34 | Female | Ruar |
| PAR11 | Dara | Recipant | 38 | Male | Ruar |
| PAR12 | Dara | Recipant | 36 | Male | Ruar |
| PAR13 | Dara | Recipant | 33 | Female | Ruar |
| PAR14 | Dara | Recipant | 29 | Female | Ruar |
| PAR15 | Dara | Recipant | 42 | Male | Ruar |
| PAR16 | Dara | Recipant | 30 | Female | Urban |
