# Supplementary material for: Implementation of a malaria prevention education intervention in Southern Ethiopia: a qualitative evaluation
Source: BMC Public Health. 2022 Sep 23;22:1811. doi: 10.1186/s12889-022-14200-x (PMC9508754; doi:10.1186/s12889-022-14200-x)
Supplement: Supplementary file 2 — Additional file 2. Socio-demographic characteristics of participants involved in the interview of barriers and facilitators of ITN use and PDAT of malaria, 2020. [file 12889_2022_14200_MOESM2_ESM.docx]

Additional file 2: Socio-demographic characteristics of participants involved in the interview of barriers and facilitators of ITN use and PDAT of malaria, 2020

| **ID** | **Place of residence** | **Age** | **Seks** | **Occupation** | **Marital status** | **educational status** | **participant** |
| --- | --- | --- | --- | --- | --- | --- | --- |
| PAR1 | urban | 50 | Male | civil servant | Married | 10th complete | parent |
| PAR2 | urban | 29 | Female | Merchant | Married | 10th complete | parent |
| PAR3 | urban | 30 | Male | Farmer | Married | grade 8 | parent |
| PAR4 | rural | 45 | Male | Farmer | married | grade 5 | parent |
| PAR5 | rural | 35 | Male | Farmer | married | Illiterate | parent |
| PAR6 | rural | 45 | Male | Farmer | married | illiterate | parent |
| PAR7 | rural | 43 | Female | Housewife | married | illiterate | parent |
| PAR8 | rural | 39 | Female | Housewife | married | illiterate | parent |
| PAR9 | rural | 41 | Male | Farmer | married | illiterate | parent |
| PAR10 | rural | 43 | Male | Farmer | married | illiterate | parent |
| PAR11 | rural | 34 | Female | Housewife | married | illiterate | parent |
| PAR12 | rural | 39 | Female | Housewife | married | illiterate | parent |
| KI1 | urban | 33 | Male | health professional | married | Degree | Key informant |
| KI2 | rural | 30 | Male | health professional | married | Diploma | Key informant |
| KI3 | rural | 35 | Female | HEW | married | Diploma | Key informant |
| KI4 | rural | 32 | Male | health professional | married | Degree | Key informant |
| KI5 | rural | 35 | Female | HEW | divorced | Diploma | Key informant |
| KI6 | rural | 40 | Male | Teacher | married | Diploma | Key informant |
| KI7 | rural | 38 | Male | Teacher | married | Degree | Key informant |
| KI8 | Semi-urban | 28 | Male | health professional | single | Diploma | Key informant |
| KI9 | rural | 25 | Male | health professional | not married | Diploma | Key informant |
